# Inhibition of MD2‐dependent inflammation attenuates the progression of non‐alcoholic fatty liver disease

**DOI:** 10.1111/jcmm.13395

**Published:** 2017-10-27

**Authors:** Yali Zhang, Beibei Wu, Hailing Zhang, Xiangting Ge, Shilong Ying, Mengwei Hu, Weixin Li, Yi Huang, Li Wang, Chao Chen, Xiaoou Shan, Guang Liang

**Affiliations:** ^1^ Chemical Biology Research Center School of Pharmaceutical Sciences Wenzhou Medical University Wenzhou Zhejiang China; ^2^ The Second Affiliated Hospital and Yuying Children's Hospital of Wenzhou Medical University Wenzhou Zhejiang China; ^3^ Department of Endocrinology The Affiliated First Hospital of Wenzhou Medical University Wenzhou Zhejiang China; ^4^ College of Life Sciences Huzhou University Huzhou Zhejiang China

**Keywords:** non‐alcoholic fatty liver disease, MD2, TLR4, inflammation, fibrosis

## Abstract

Non‐alcoholic fatty liver disease (NAFLD) can progress to the more serious non‐alcoholic steatohepatitis (NASH), characterized by inflammatory injury and fibrosis. The pathogenic basis of NAFLD progressing to NASH is currently unknown, but growing evidence suggests MD2 (myeloid differentiation factor 2), an accessory protein of TLR4, is an important signalling component contributing to this disease. We evaluated the effectiveness of the specific MD2 inhibitor, L6H21, in reducing inflammatory liver injury in a relevant high‐fat diet (HFD) mouse model of NASH and in the palmitic acid (PA)‐stimulated human liver cell line (HepG2). For study, genetic knockout (MD2^−/−^) mice were fed a HFD or control diet for 24 weeks, or wild‐type mice placed on a similar diet regimen and treated with L6H21 for the last 8 or 16 weeks. Results indicated that MD2 inhibition with L6H21 was as effective as MD2 knockout in preventing the HFD‐induced hepatic lipid accumulation, pro‐fibrotic changes and expression of pro‐inflammatory molecules. Direct challenge of HepG2 with PA (200 μM) increased MD2‐TLR4 complex formation and expression of pro‐inflammatory and pro‐fibrotic genes and L6H21 pre‐treatment prevented these PA‐induced responses. Interestingly, MD2 knockout or L6H21 increased expression of the anti‐inflammatory molecule, PPARγ, in liver tissue and the liver cell line. Our results provide further evidence for the critical role of MD2 in the development of NASH and conclude that MD2 could be a potential therapeutic target for NAFLD/NASH treatment. Moreover, the small molecule MD2 inhibitor, L6H21, was an effective and selective investigative agent for future mechanistic studies of MD2.

## Introduction

NAFLD is an emerging global health problem, which is characterized by excess fat accumulation in the liver (steatosis) due to causes other than alcohol. It can progress to become the more serious NASH, a state in which steatosis is combined with inflammatory injury and fibrosis that can lead to end‐stage liver cirrhosis 1, 2. The development and progression of NAFLD to NASH remain unclear, but obesity (*i.e*. metabolic syndrome) and insulin resistance are likely key factors in the pathogenesis. One prevalent hypothesis is the ‘Two‐hit theory’ that considers hepatic steatosis as first hit, rendering the liver more susceptible to a second hit(s) (such as with inflammation, ROS and ER stress), thereby driving NAFLD progression towards NASH or even to cirrhosis 3.

Increasing evidence implicates inflammation as a critical factor in the progression of NAFLD to NASH 4, 5, 6. Reports obtained from different experimental models over the past several years indicate that the occurrence and severity of NASH appear to be dependent on activation of toll‐like receptor 4 (TLR4) 7, 8, 9, 10, 11, 12. TLR4 is a pattern recognition receptor of the immune system, which can be activated by microbial products (*i.e*. LPS) or endogenous free fatty acids (FFAs), producing a host of pro‐inflammatory cytokines. It is currently believed that the inflammatory injury of NASH is likely attributed to TLR4 activation by pathogenic (*e.g*. LPS) and/or non‐pathogenic ligands (*e.g*. FFAs), triggering recruitment of adaptor protein myeloid differentiation factor 88 (MyD88), which leads to activation of signalling cascades (*i.e*. NF‐κB and MAPKs) for generation of pro‐inflammatory molecules. Interestingly, Sharifnia *et al*. 13 observed up‐regulated TLR4 expression in liver, elevated plasma FFAs as well as lipopolysaccharide (LPS) in a large cohort of obese human patients with NASH, suggesting a positive feedback regulation of TLR4.

MD2, the extracellular glycoprotein accessory protein of TLR4, appears to be an important component in the inflammatory responses of NASH. Findings indicate that the genetic deletion of MD2 significantly reduces the inflammatory injury and fibrosis in a methionine‐choline deficiency mouse model of NASH 14, further supporting the MD2‐TLR4 pathway as an underlying pathogenic mechanism. MD2 is expressed on many cell types in the liver including hepatocytes, stellate cells and Kupffer cells 14, but precisely how activation of MD2‐TLR4 occurs under conditions of NAFLD/NASH remains unclear. At least for LPS and FFA signalling, LPS is required to bind MD2, which in turn binds to the extracellular domain of TLR4, resulting in MD2‐TLR4‐MyD88 complex formation and activation 15, 16. Thus, MD2 has a broad and significant driving acute and chronic inflammatory responses 17, 18, 19 and is considered to be a potential therapeutic target for several inflammatory diseases 20, 21, 22. Inhibitors of MD2 are highly effective anti‐inflammatory agents in LPS‐caused acute inflammatory diseases 20 and FFA‐induced cardiac inflammatory injury 16. Recently, our group has designed and synthesized a specific MD2 inhibitor, L6H21, which also protects against diabetes‐related renal injury through blocking the inflammatory response 19.

Moreover, the downstream signals of MD2/TLR4 appear to be modulated through cross‐talk with PPARγ (peroxisome proliferator‐activated receptor gamma), a member of the nuclear receptor superfamily which can be activated by a variety of ligands, for example eicosanoids and fatty acids 23. Several reports indicate that PPARγ agonists are protective against NAFLD/NASH by reducing inflammation, possibly through inhibiting production of inflammatory cytokines 24, 25, 26, 27, 28, 29. MEKs, the activator of MAPKs (a key signalling target of MD2/TLR4), is reported to directly interact with PPARγ, resulting in down‐regulation of PPARγ through nuclear export 30, suggesting an intriguing mechanism of promoting pro‐inflammatory activity through suppressing PPARγ's anti‐inflammatory activity.

In this study, we evaluated the effectiveness of the specific MD2 inhibitor 21, L6H21, in reducing inflammatory liver injury in a HFD mouse model of NASH and in the PA‐stimulated human liver cell line. Additionally, we explored the regulation of PPARγ by the MD2‐TLR4 complex in the context of inflammatory injury in NASH. The results indicated that MD2 inhibition with L6H21 was as effective as MD2 knockout in preventing the progression of inflammatory injury and fibrosis in the liver with NASH. L6H21 also effectively prevented the PA‐induced MD2/TLR4 complex formation as well as the inflammatory responses in liver cell line. Interestingly, MD2 knockout or L6H21 increased PPARγ expression in liver tissue and liver cell line. We conclude the MD2 is a key signalling component for the inflammatory injury and fibrosis in NASH.

## Materials and methods

### Reagents

PA (C16:0), bovine serum albumin (BSA) and Masson's trichrome were purchased from Sigma‐Aldrich (St. Louis, MO, USA). Antibodies for collagen‐IV (COL‐IV), TLR4, MD2 and anti‐rabbit and antimouse horseradish peroxidase (HRP)‐conjugated secondary antibody were obtained from Santa Cruz Technology (Santa Cruz, CA, USA). Antibodies for tumour necrosis factor (TNF)‐α, PPARγ and GAPDH were from Cell Signaling Technology (Danvers, MA, USA). Antibody for α‐SMA was purchased from Abcam (Cambridge, MA, USA). RIPA lysis buffer was purchased from Boster Biological technology (Wuhan, China).

L6H21 is an MD2 specific small molecule inhibitor designed, synthesized and purified to 99% by our group.^21^ L6H21 was dissolved in dimethyl sulfoxide (DMSO) for *in vitro* experiments and in 1% sodium carboxyl methyl cellulose (CMC‐Na) for *in vivo* experiments.

The assay kits for triglyceride (TG), total cholesterol (TCH), high density lipoprotein‐cholesterol (HDL‐c), low density lipoprotein‐cholesterol (LDL‐c) and hydroxyproline were purchased from Nanjing Jiancheng Bioengineering Institute (Nanjing, China). Oil Red O solution was obtained from Nanjing Jiancheng Bioengineering Institute. Haematoxylin–eosin (H&E) was purchased from Beyotime (Nantong, China). TRIZOL and M‐MLV Platinum SYBR Green qPCR SuperMix‐UDG kit were purchased from Invitrogen (Carlsbad, CA, USA). A (HFD, 60 kcal.% fat, 20 kcal.% protein and 20 kcal.% carbohydrate, Cat. #MD12033) and a standard rodent diet (containing 10 kcal.% fat, 20 kcal.% protein and 70 kcal.% carbohydrate Cat. #MD12031) were purchased from Mediscience Diets Co. LTD (Yangzhou, China).

### Cell culture and treatment

Human hepatocellular carcinoma cell line HepG2 (Shanghai Institute of Biochemistry and Cell Biology, Shanghai, China) was maintained in RPMI‐1640 (Gibco, Eggenstein, Germany) with 10% Foetal Bovine Serum, 100 mg/ml streptomycin and 100 U/ml penicillin at 37°C with 5% CO_2_. Stock solutions of 5 mM PA/5% BSA were heated for 15 min. at 55°C and cooled to room temperature to obtain the PA‐BSA complex. The PA‐BSA mixture was added to serum‐containing cell culture medium to final concentration of 200 μM. HepG2 cells were treated with different concentrations of L6H21, followed with incubation with 200 μM PA for 24 hrs. HepG2 cells maintained in culture medium containing 5% BSA served as a control.

### Mouse study

#### Animal care

All mouse study protocols used were approved by the Wenzhou Medical University Animal Policy and Welfare Committee. Forty male C57BL/6 mice aged 8 weeks were obtained from the Animal Center of Wenzhou Medical College (Wenzhou, China). Sixteen male MD2^−/−^ mice with C57BL/6 background (B6.129P2‐Ly96 KO) were introduced from Riken BioResource Center of Japan (Tsukuba, Ibaraki, Japan). Mice were housed at 22°C with a 12:12‐hr light–dark cycle. All animal care and experimental procedures complied with the ‘The Detailed Rules and Regulations of Medical Animal Experiments Administration and Implementation’ (Order No. 1998‐55, Ministry of Public Health, China) and were approved by the Wenzhou Medical University Animal Policy and Welfare Committee.

#### High‐fat diet

After an acclimatization period of 1 week, male C57BL/6 mice were randomly divided into five weight‐matched groups (in each group *n* = 8). Eight mice were fed with standard rodent diet served as a normal control group (CON), while the remaining 32 mice were fed with HFD for 24 weeks. From 9th to 24th week, eight HFD‐fed mice were treated with L6H21 (20 mg/kg) once every 2 days by oral gavage for 16 weeks (L6H21‐20 HFD group, *n* = 8, for prevention). From 17th to 24th week, 16 HFD‐fed mice were treated with L6H21 (10 mg/kg or 20 mg/kg, respectively) once every 2 days by oral gavage for 8 weeks (HFD L6H21‐10 group, HFD L6H21‐20 group, *n* = 8 per group, for treatment). Mice in the CON and HFD alone groups were gavage with vehicle (1% CMC‐Na solution) only (*n* = 8 per group). Sixteen gender‐ and age‐matched male MD2^−/−^ mice were randomly divided into control (MD2^−/−^ CON) and HFD (MD2^−/−^ HFD) group (*n* = 8 per group). MD2^−/−^ CON group feed with standard rodent diet and MD2^−/−^ HFD group received a HFD diet for 24 weeks. Body weight was monitored weekly during the feeding/treatment period. Twenty‐four weeks after the first treatment, the mice were killed under anaesthesia. The body weight was recorded, and serum was collected after blood samples being centrifuged at 4°C at 845 g for 15 min. Liver tissues were harvested for subsequent analyses.

### Pathological staining analysis

Livers were fixed in a 4% paraformaldehyde solution and embedded in paraffin. After deparaffinization and rehydration, liver sections (5 μm) were stained with H&E viewed by a fluorescence microscope (×200; Nikon, Tokyo, Japan), or Masson's trichrome viewed by a fluorescence microscope (×400; Nikon). NAFLD activity score (steatosis (0–2), inflammation (0–2) and ballooning degeneration (0–2)) were performed by a blinded pathologist. The ratio of fibrosis was qualified by an image analysis on Masson's trichrome staining of the liver sections of the eight mice in each group. At ×400 magnification, the Image‐pro Plus (Media Cybernetics Inc., Silver Spring, MD, USA) software was used to quantify the amount of liver tissue (red signal) and collagen (blue signal) inside the liver tissue of 10 randomly chosen frames. The ratio of collagen to liver tissue was recorded for each specimen.

### Oral glucose tolerance tests (OGTT)

Mice were fasted overnight followed by oral gavage glucose (1.0 g/kg body weight). Blood samples were collected from tail veins at 0, 15, 30, 60 and 120 min. after glucose gavage, and blood glucose levels were measured using the blood glucometer (One Touch; Johnson & Johnson, New Brunswick, NJ, USA).

### Oil red O staining

To determine the TG content in liver tissue, liver tissues were embedded in OCT and stored at −80°C. Liver sections (20 μm thick) were incubated with 60% filtered Oil Red O solution for 3 min. at room temperature followed by haematoxylin staining for 5 min. They were viewed by fluorescence microscope (×400; Nikon). At ×400 magnification, the TG and liver section area of ten random fields were measured quantitatively with Image‐pro Plus software (Media Cybernetics Inc.). The ratio of TG to liver tissue was recorded for each specimen.

### Immunohistochemistry

The 5 μm liver sections were subjected to deparaffinization and rehydration followed by treatment with 3% H_2_O_2_ for 10 min. After incubating with 1% BSA in phosphate buffer for 30 min., liver sections were incubated with primary antibodies for α‐SMA (1:200), PPARγ (1:200) and TNF‐α (1:200) overnight at 4°C. Then, the tissues were incubated with a 1:200 dilution of HRP‐conjugated secondary antibody for 1 hr at room temperature. The nuclei were counter‐stained with haematoxylin for 5 min. The sections were viewed by a fluorescence microscope (×400; Nikon). At ×400 magnification, the area of the staining signal (α‐SMA, PPARγ or TNF‐α) of ten random fields was measured using the Image‐pro Plus software (Media Cybernetics Inc.). The percentage expression of α‐SMA, PPARγ or TNF‐α was recorded for each specimen.

### Immunoprecipitation

HepG2 cells were treated with PA for 24 hr after L6H21 pre‐treatment. Prepared cell protein samples or liver tissue samples were incubated with anti‐TLR4 antibody at 4°C overnight and then precipitated with protein G‐Sepharose beads at 4°C for 3 hrs. The MD2 level in the beads was further detected by Western Blot using an anti MD2 antibody.

### Circulating lipid levels

Whole blood was collected from the anaesthetized animals. The serum was prepared by centrifugation of blood at 3000 rpm for 15 min. at 4°C and stored at −80°C until analysis. The TG, TCH, HDL‐c, LDL‐c and hydroxyproline levels in serum were measured using commercial assay kits (Nanjing Jiancheng Bioengineering Institute).

### Liver lipid levels

For TG and TCH measurement, lipids in the homogenate from 200 mg of liver sample were extracted with 3 ml of ethanol‐acetone (1:1) thrice. After overnight sedimentation, samples were centrifuged at 4°C, and the supernatant was obtained for analysis using commercially available kits (Applygen Technologies Inc., Beijing, China).

### Western blot analysis

Frozen tissue samples and cell protein samples were lysed in RIPA lysis buffer (1% NP‐40, 1% Sodium deoxycholate, 0.1% SDS). The cell lysates were centrifuged at 15616 g for 10 min. at 4°C, and the supernatant was collected. Bradford Assay was used to determine the protein concentration. After normalized, equal amounts of proteins were subjected to 10% SDS‐PAGE and transferred to a polyvinylidene fluoride (PVDF) membrane (Bio‐Rad Laboratories, Hercules, CA, USA). The membranes were pre‐incubated for 1.5 hrs at room temperature with 5% non‐fat dry milk in TBST (Tris‐buffered saline containing 0.05% Tween 20) and incubated with specific primary antibodies overnight at 4°C (the dilution for antibodies is TBS‐T; the dilution rate of COL‐IV, MD2 and TLR4 antibodies is 1:300; the dilution rate of PPARγ and GAPDH antibodies is 1:1000). After being washed in TBST, the membranes were incubated with a 1:3000 dilution of HRP‐conjugated secondary antibody for 1 hr at room temperature, and visualized using enhanced chemiluminescence reagents (Bio‐Rad Laboratories, Hercules, CA, USA).

### Reverse transcription and real‐time quantitative PCR (RT‐qPCR)

Total RNA was isolated from cells or liver tissues (40–50 mg) using TRIZOL. Reverse transcription and quantitative PCR were performed using an M‐MLV Platinum SYBR Green qPCRSuperMix‐UDG kit following the manufacturer's instructions. Primers for α‐SMA, transforming growth factor (TGF)‐β, COL‐IV, COL‐I, matrix metalloproteinase (MMP)‐9, TNF‐α, IL‐1β, vascular cell adhesion molecule (VCAM)‐1, intercellular cell adhesion molecule (ICAM)‐1, monocyte chemotactic protein (MCP)‐1 and β‐actin were synthesized by Invitrogen (Shanghai, China). The relative amount of each gene was normalized to the amount of β‐actin. The primer sequences used are shown in Table S1.

### Data analysis

All experiments were performed at least in triplicate. Results were presented as means ± S.E.M.s. The significance was analysed with Prism 5.0 (GraphPad Software, La Jolla, CA, USA) by Student's *t*‐test. It was considered significant when *P* value was < 0.05 (*P < 0.05, **P < 0.01 and ***P < 0.001).

## Results

### MD2 inhibition or knockout prevents liver lipid accumulation

**Figure 1:**
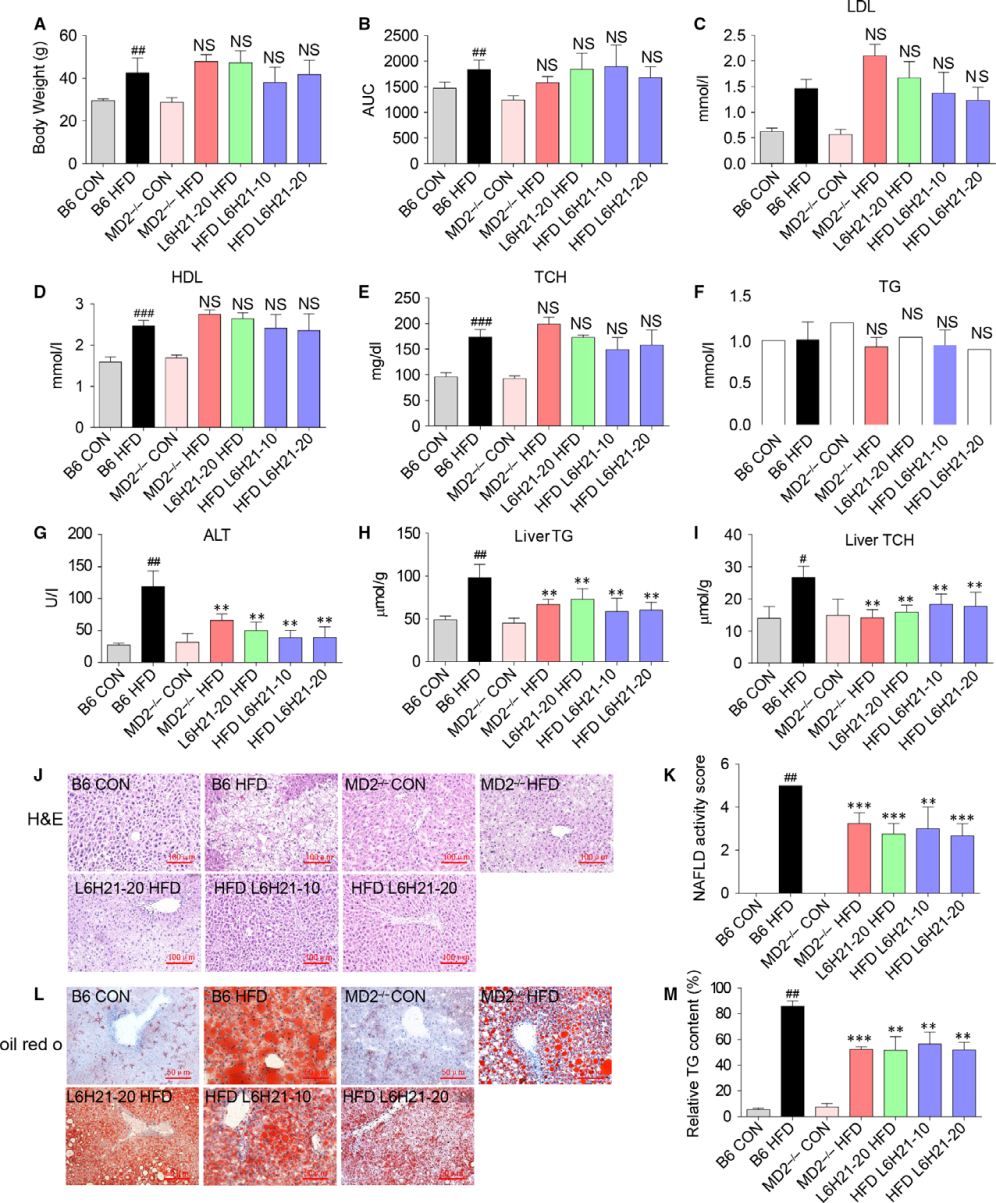
Myeloid differentiation factor 2 (MD2) inhibition or MD2 knockout reduced liver injury and lipid accumulation associated with HFD. Male C57BL/6 mice, at 8 weeks of age, were fed a HFD or control low fat diet for 24 weeks and treated with L6H21 20 mg/kg once every 2 days by oral gavage for last 16 weeks (L6H21‐20 HFD), or treat with L6H21 10 mg/kg or 20 mg/kg for last 8 weeks (HFD L6H21‐10, HFD L6H21‐20, respectively). Age‐ and gender‐matched MD2^−/−^ mice with C57BL/6 background were feed with HFD for 24 weeks (MD2^−/−^ HFD). (**A**) Body weight was monitored weekly during the feeding/treatment period. (**B**) AUC of OGTT. (**C**) LDL‐c and (**D**) HDL‐c (**E**) TCH, (**F**) TG, in serum were detected as described in the Materials and methods. (**G**) Liver function was determined by detecting the ALT level in serum. (**H**) TG and (**I**) TCH in liver tissue were detected as described in the Materials and methods. Representative histopathological changes (**J**) in liver tissue detected with H&E staining (×200), and lipid accumulation (**L**) indicated by oil red o staining (×400). (**K**) NAFLD activity score (steatosis, inflammation and ballooning degeneration) performed by a blinded pathologist at study end. (**M**) Quantification for the percentage of relative TG content determined by oil red O staining in panel **L**. Data are shown as mean ± S.E.M. (*n* = 8, ^##^ *P* < 0.01, ^###^ *P* < 0.001, *versus* B6 CON group, NS, no significant difference *versus* B6 HFD group; **P < 0.01, ***P < 0.001, *versus* B6 HFD group).

The requirement for MD2 in the HFD‐induced liver injury was investigated using the specific pharmacological MD2 inhibitor, L6H21 21 or genetic knockout mice (MD2^−/−^). Compared to the control diet mice (B6 CON), HFD (B6 HFD) increased the body weight, elevated area under the curve (AUC) of OGTT and increased serum level of LDL, HDL and TCH (Fig. 1A–E), but not TG (Fig. 1F). The elevated body weight and serum lipid profile were associated with enlarged hepatocytes (H&E stain) and significant lipid accumulation (Oil Red O) (Fig. 1J and L). Neither MD2^−/−^ mice nor mice treated with L6H21 blocked elevated levels of body weight, AUC of OGTT, LDL, HDL and TCH as that of the HFD group (Fig. 1A–E), indicating that MD2 blockage did not affect the hyperlipidemia profile in HFD mouse blood. To determine the effect of MD2 blockage on liver function, we measured the serum ALT level. Compared to the control diet mice (B6 CON), HFD (B6 HFD) showed markedly increased serum ALT level. However, it was effectively reduced in MD2^−/−^ HFD mice and HFD mice treated with L6H21 at 10 or 20 mg/kg. (Fig. 1G). Meanwhile, MD2 blocking significantly attenuated HFD‐induced lipid accumulation in liver, which was demonstrated by detecting the TG (Fig. 1H) and TCH (Fig. 1I) content in liver tissue. Both H&E staining of liver sections (Fig. 1J), and NAS (Fig. 1K), the quantitative result of H&E staining, showed HFD‐induced liver damage attenuated in MD2^−/−^ HFD mice and HFD mice treated with L6H21 at 10 or 20 mg/kg. Oil red O staining (Fig. 1L) and quantitative result of Oil red O staining (Fig. 1M) indicated HFD group had higher TG content compared with CON group, which was decreased in MD2^−/−^ HFD mice and HFD mice treatment. MD2 knockout or L6H21 administration significantly reduced the HFD‐induced hepatic steatosis, indicating that MD2 deficiency may protect against liver injury. Unfortunately, we did not measure the liver size and weight the liver tissues when we collected the liver sample, which may be a limitation of this work.

### MD2 inhibition or knockout protects against HFD‐induced liver fibrosis

**Figure 2:**
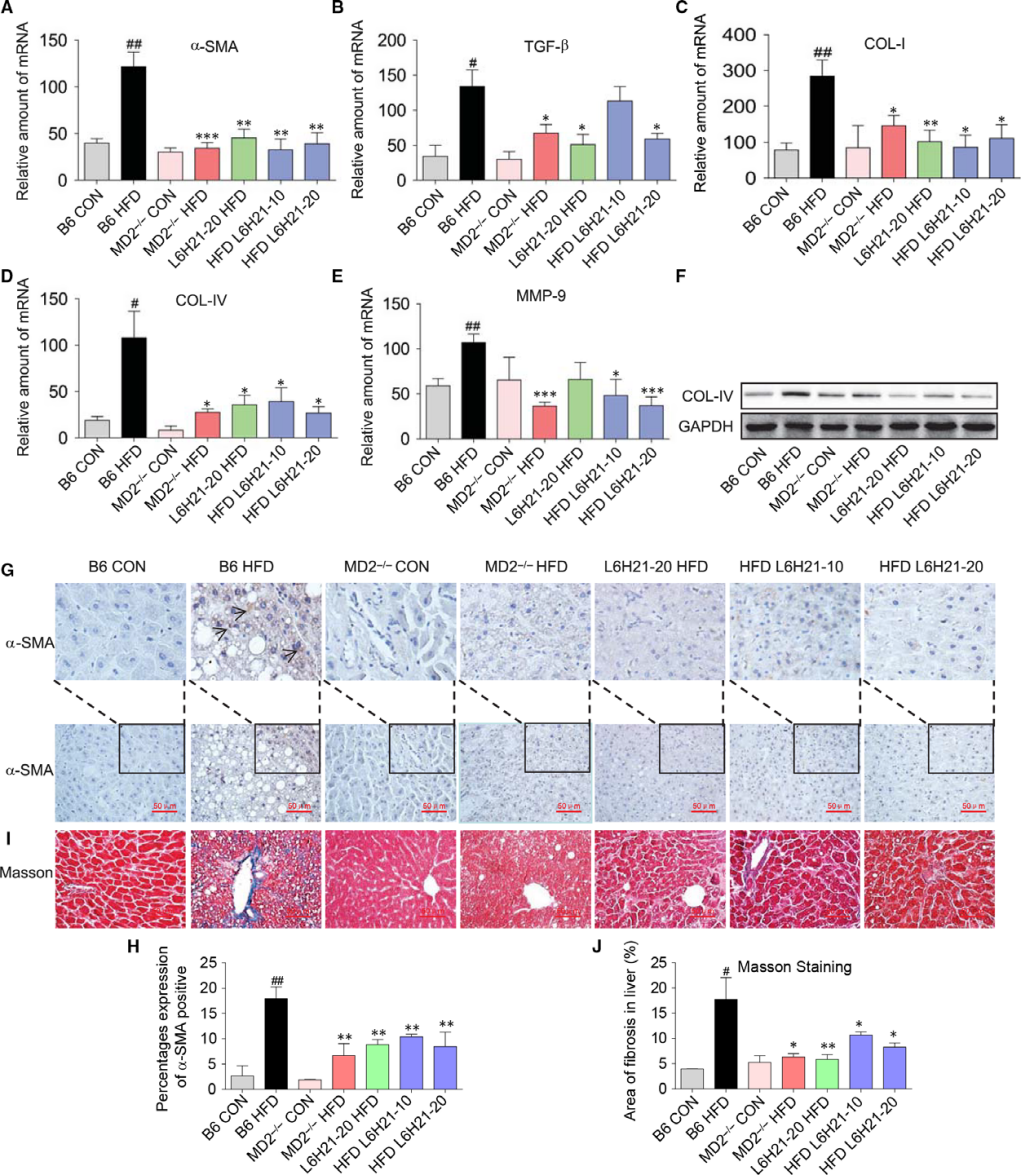
Myeloid differentiation factor 2 (MD2) inhibition or MD2 knockout reduced liver fibrosis associated with HFD. Experimental groups of mice are as described in Figure 1 (Materials and methods). (**A**–**E**) The mRNA expression of the fibrosis‐associated genes was measured by RT‐qPCR from liver tissue; β‐actin served as internal control: (**A**) α‐SMA, (**B**) TGF‐β, (**C**) COL‐I, (**D**) COL‐IV and (**E**) MMP‐9. (**F**) Total proteins extracted from liver tissues were subjected to Western blot analysis for COL‐IV level; (**G**) α‐SMA immunostaining (×400) and (**I**) Masson's Trichrome (×400) staining were used as indices of liver fibrosis. (**H**) Quantification for percentages expression of α‐SMA protein in panel **G**. (**J**) Quantification for the area of fibrosis in liver tissues detected by Masson staining in panel **I**. Data are shown as mean ± S.E.M. (*n* = 8 per group, ^#^ *P* < 0.05, ^##^ *P* < 0.01, *versus* B6 CON group; *P < 0.05, **P < 0.01, ***P < 0.001, *versus* B6 HFD group).

A key challenge in human NAFLD is its progression to fibrosis and cirrhosis. As expected, the expression of genes associated with fibrosis, that is, α‐SMA, TGF‐β, COL‐I, COL‐IV and MMP‐9, was markedly increased in liver tissue of the B6‐HFD group (Fig. 2A–E). In mice treated with L6H21 or MD2^−/−^ mice, the HFD‐induced increases of these genes were prevented (Fig. 2A–E). Moreover, protein expression of COL‐IV in liver tissue was up‐regulated in the B6 HFD mice, and blocked by L6H21 treatment or in MD2^−/−^ mice (Fig. 2F). Consistent with the development of hepatic fibrosis, α‐SMA immunohistochemistry staining revealed increased expression of α‐SMA in liver tissue of the B6 HFD mice; however, the increase was significantly reduced with L6H21 treatment or MD2 knockout (Fig. 2G and H). Masson staining for collagen confirmed that in the B6 HFD mice (Fig. 2I) and the quantitative result of Masson staining was shown in Fig. 2J, indicating that the increased collagen deposition in liver tissue was prevented by L6H21 treatment or MD2 knockout (Fig. 2I). The results indicated that MD2 is a likely regulator in the progression of HFD‐associated liver fibrosis.

### MD2 inhibition or knockout increases expression of PPARγ in liver

**Figure 3:**
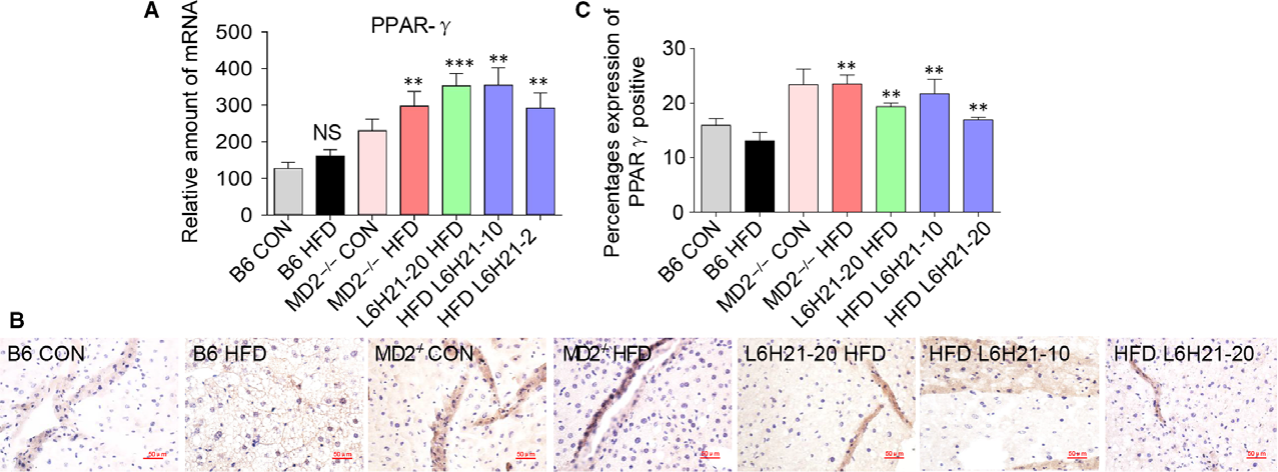
Myeloid differentiation factor 2 (MD2) inhibition or MD2 knockout increased expression of PPAR‐γ. Experimental groups of mice are as described in Figure 1 (Materials and methods). (**A**) The expression of PPARγ was analysed by RT‐qPCR using β‐actin mRNA as internal control. (**B**) Representative image of immunohistochemical staining of PPARγ in liver tissue sections; *n* = 8; (×400) magnification. (**C**) Quantification for the percentages expression of PPARγ in liver tissues in panel **B**. Data are shown as mean ± S.E.M. (*n* = 8 per group, NS, no significant difference *versus* B6 CON group, *P < 0.05, **P < 0.01, ***P < 0.001, *versus* B6 HFD group).

Evidence implicates PPARγ in contributing to the pathogenesis of NAFLD 23, 24, 25, 26. We next explored whether PPARγ's involvement was associated with the MD2/TLR4 signalling pathway. HFD alone did not alter the expression level of PPARγ mRNA; however, L6H21 treatment or MD2 knockout significantly increased the PPARγ expression compared to the B6 HFD group (Fig. 3A). Consistently, PPARγ immunohistochemistry as well as the quantitative analysis showed that there was no significant difference between CON and HFD group, while L6H21 treatment or MD2 knockout enhanced hepatic PPARγ level compared to B6 HFD group (Fig. 3B and C). These results indicated that MD2/TLR4 signalling modulated the levels of PPARγ in the mouse model.

### MD2 inhibition or knockout protects against HFD‐induced liver inflammation

**Figure 4:**
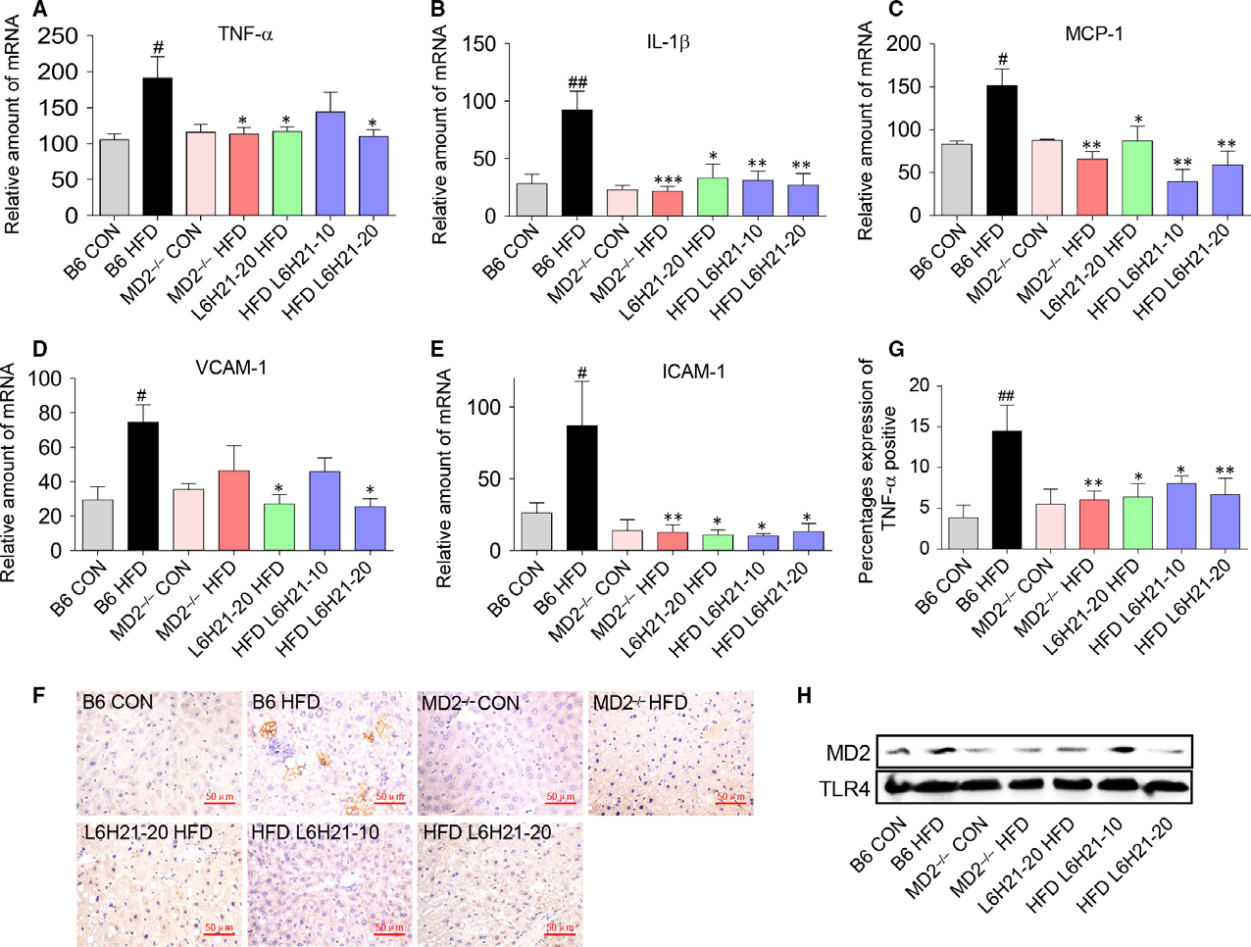
Myeloid differentiation factor 2 (MD2) inhibition or MD2 knockout reduced liver inflammation associated with HFD. Experimental groups of mice are as described in Figure 1 (Materials and methods). (**A**‐**E**) The mRNA expression of key inflammatory molecules in liver tissue was analysed by RT‐qPCR using β‐actin mRNA as the internal control: (**A**) TNF‐α, (**B**) IL‐1β, (**C**) MCP‐1, (**D**) VCAM‐1 and (**E**) ICAM‐1. (**F**) Representative immunohistochemical detection of TNF‐α in liver tissue sections; *n* = 8; (×400) magnification. (**G**) Quantification for the percentages expression of TNF‐α protein in panel **F**. Data are shown as mean ± S.E.M. (*n* = 8 per group, ^#^ *P* < 0.05, ^##^ *P* < 0.01, *versus* B6‐CON group, *P < 0.05, **P < 0.01, *versus* B6‐HFD group). (**H**) Immunoprecipitation assay shows that MD2 knockout or inhibition by L6H21 significantly reduced the formation of TLR4‐MD2 complex in liver tissue induced by HFD.

As inflammation is central in driving the progression of NAFLD, we quantified key inflammation‐associated genes, cytokines TNF‐α, IL‐1β and MCP‐1 and genes of adhesion molecules, that is VCAM‐1 and ICAM‐1. As expected, HFD significantly increased expression of cytokines and adhesion molecules in liver tissue of B6 HFD mice (Fig. 4A–E). The treatment with L6H21 or MD2 knockout prevented the HFD‐associated increase in the inflammatory genes, thereby reducing the severity of the hepatic inflammatory state (Fig. 4A–E). Consistent with the mRNA data, TNF‐α immunohistochemistry revealed increased expression in the liver tissue of B6 HFD mice, and L6H21 treatment or MD2 knockout prevented the increased TNF‐α expression (Fig. 4F). The result was further confirmed by the quantitative analysis (Fig. 4G). Further, co‐precipitation analysis of liver tissues indicated an increased MD2‐TLR4 complex formation in HFD treated mice, while the increase was significantly reduced with L6H21 treatment or MD2 knockout (Fig. 4H).

### L6H21 improves PA‐induced inflammatory responses in HepG2 cells

**Figure 5:**
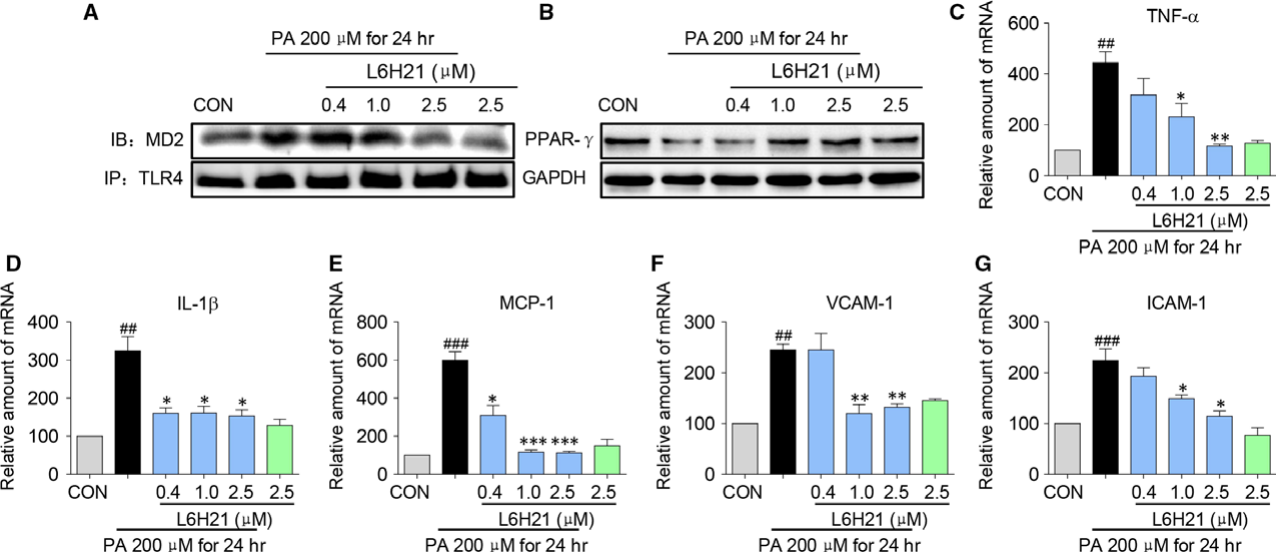
L6H21 inhibited PA‐induced inflammatory response in liver cells. HepG2 cells were pre‐treated with concentrations of L6H21 (0.4, 1.0 and 2.5 μM) for 1 hr, incubated with PA (200 μM) for 24 hrs and evaluated as follows: (**A**) Co‐immunoprecipitation of TLR4 and MD2; shown is representative blot for MD2; shown is representative of three determinations. (**B**) Total protein of PPARγ; shown is representative blot from three determinations. HepG2 cells were pre‐treated with concentrations of L6H21 (0.4, 1.0 and 2.5 μM) for 1 hr, incubated with PA (200 μM) for 24 hrs, and key inflammatory molecules were quantified by RT‐qPCR analysis using β‐actin mRNA as the internal control: (**C**) TNF‐α, (**D**) IL‐1β, (**E**) MCP‐1, (**F**) VCAM‐1 and (**G**) ICAM‐1. Data are expressed as the mean ± S.E.M. from three to five independent experiments; (^#^ *P* < 0.05, ^##^ *P* < 0.01, ^###^ *P* < 0.001, *versus* CON group, *P < 0.05, **P < 0.01, ***P < 0.001, *versus* PA treatment alone).

We next investigated whether the protective effects of L6H21 observed in the *in vivo* mice model were attributed to a direct effect on hepatocytes. We previously found that PA induces MD2/TLR4‐dependent inflammation in macrophage and cardiomyocytes *via* directly binding MD2 16. For this study, we pre‐treated HepG2 cells with L6H21 (0.4, 1.0 or 2.5 μM) for 1 hr, followed by stimulation with PA (200 μM) for 24 hrs. The increased MD2‐TLR4 complex formation was consistent with activation of this signalling complex. Results indicated that PA stimulated MD2‐TLR4 complex formation; however, L6H21 pre‐treatment reduced the PA‐induced complex formation in a dose‐dependent manner (Fig. 5A). We next determined the signalling activities downstream of MD2/TLR4. Results from our mouse model studies indicated that in response to a HFD, MD2 deficiency promoted PPARγ expression in liver tissue. In HepG2 cells, the stimulation with PA significantly decreased expression of PPARγ as determined by Western blot analysis of total cell lysate (Fig. 5B), whereas the pre‐treatment with L6H21 prevented the PA‐induced decrease (Fig. 5B). HepG2 cells stimulated with PA for 24 hrs also presented with significant increases of a panel of inflammatory genes encoding cytokines TNF‐α, IL‐1β and MCP‐1, as well as adhesion molecules VCAM‐1 and ICAM‐1 (Fig. 5C–G). Importantly, when HepG2 cells were pre‐treated with L6H21, the PA‐induced increased expressions of these pro‐inflammatory genes were effectively inhibited, particularly at the high doses (Fig. 5C–G). Taken together, these results indicated that PA induced decreased PPARγ expression with concomitant increases in several pro‐inflammatory genes, which were prevented with inhibition of MD2 by L6H21. Further, findings suggest that MD2 is likely a critical pathway functioning the hepatocytes.

### L6H21 prevented PA‐induced expression of pro‐fibrotic genes in HepG2 cells

**Figure 6:**
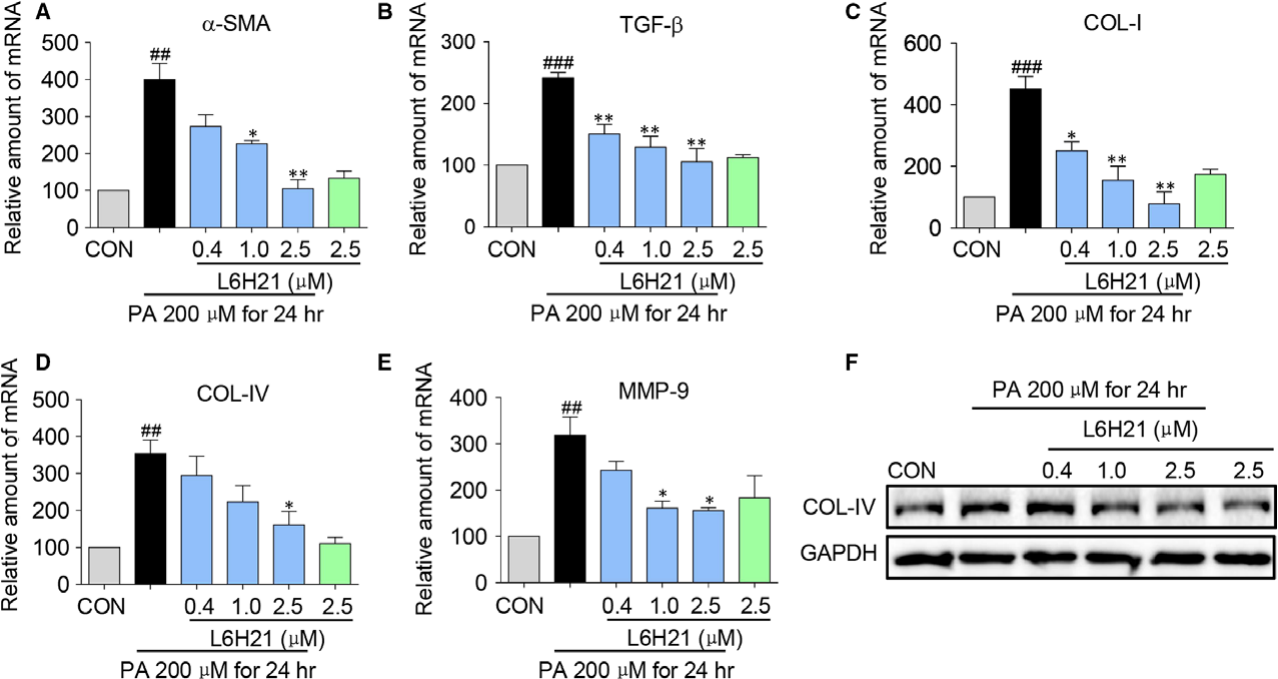
L6H21 inhibited PA‐induced fibrosis‐related gene and protein expression in liver cells. HepG2 cells were pre‐treated with a series concentration of L6H21 (0.4, 1.0, 2.5 μM) for 1 hr, incubated with PA (200 μM) for 24 hrs, and the mRNA levels of fibrosis‐related genes (**A**) α‐SMA, (**B**) TGF‐β, (**C**) COL‐I, (**D**) COL‐IV and (**E**) MMP‐9 were quantified by RT‐qPCR analysis using β‐actin mRNA as the internal control. Data shown are the mean ± S.E.M. from three to five independent experiments; (^#^ *P* < 0.05, ^##^ *P* < 0.01, ^###^ *P* < 0.001, *versus* CON group, *P < 0.05, **P < 0.01, *versus* PA treatment alone). (**F**) Western blot detection of COL‐IV from cell lysate; shown is representative of three determinations.

We next evaluated the potential protective effects of L6H21 on liver fibrosis *in vitro*. HepG2 cells were pre‐treated with L6H21 (0.4, 1 and 2.5 μM) for 1 hr, and then exposed to PA (200 μM) for 24 hrs. Results indicated that PA induced marked increases of genes associated with fibrosis (α‐SMA, TGF‐β, COL‐IV, COL‐I and MMP‐9) (Fig. 6A–E). With L6H21 pre‐treatment, the PA‐induced increased expressions of the pro‐fibrotic genes were decreased in a dose‐dependent manner (Fig. 6A–E). Moreover, PA significantly increased protein expression of collagen‐IV, with L6H21 pre‐treatment preventing the increase, particularly at the higher dose (Fig. 6F). These *in vitro* results provide further corroboration of the MD2‐TLR4 signalling in the hepatic inflammatory injury observed in the HFD mouse model.

## Discussion

The progression of NAFLD to NASH is a burgeoning health problem worldwide, which is characterized by hepatic lipid accumulation and inflammatory injury. Several pieces of evidence indicate that a crucial pathogenic basis of the disease is attributed to the MD2‐TLR4 complex of the immune system. In this study, we used the HFD mouse model, shown to provide similar metabolic context as in human NASH 31, to evaluate the effectiveness of the specific MD2 inhibitor, L6H21, in reducing inflammatory liver injury. We found that MD2 inhibition with L6H21 (both prevention and treatment) was as effective as MD2 knockout in preventing the progression of inflammatory injury and fibrosis in the liver with NASH, which was associated with inhibition of the MD2‐TLR4 complex formation as well. Consistently, L6H21 was effective in inhibition of the PA‐stimulated inflammatory responses and MD2‐TLR4 complex formation in HepG2 cells. Our results provide new evidence that the MD2‐TLR4 signalling complex is crucial in driving the inflammatory and fibrosis of NASH.

Although the specific ligands under conditions of NAFLD‐NASH in activating the MD2‐TLR4 have yet to be identified, our data obtained from HepG2 cells indicated that PA is likely one contributing TLR4 ligand. Similarly, Sharifinia *et al*. 13, found that PA stimulated NF‐κB activation in primary hepatocytes and an immortalized hepatic cell line. Free fatty acids (FFAs) are present in the obese state, and increased levels of FFAs, such as PA (C16:0) and stearic acid (C18:0), have been reported in patients with NASH 13. MD2 and TLR4 are expressed by other resident cells in the liver, such as kupffer cells, fibroblasts, stellate cells and endothelial cells and therefore can be activated and contribute to the local inflammation. Moreover, we 32 and others 33, 34 reported that saturated FFAs activate inflammatory and innate immune responses in a variety of cells. However, we cannot rule out contribution by other FFA ligands or LPS in activation of the MD2‐TLR4 complex in signalling the inflammatory injury in NASH. Recent evidence indicates that NASH patients have elevated plasma levels of LPS which is attributed to increased bacterial overgrowth, causing a leaky intestinal barrier function 11, 13. These findings suggest that hepatic cells might be exposed to not only FFAs but also intestinal microbial products capable of activating the MD2‐TLR4 complex. Nevertheless, as the pattern recognition receptor of LPS, MD2/TLR4 is also responsible for the inflammatory response induced by LPS in liver. Thus, inhibition of MD2 may be an interesting strategy for the treatment of NASH by blocking FFA‐ or LPS‐induced inflammation in HFD liver.

Our HFD mouse model produced inflammatory and fibrotic changes characteristic of human steatohepatitis and metabolic profile. Specifically, the mouse liver tissue showed increased accumulated lipids, inflammatory molecules and molecules associated with tissue remodelling. Moreover, unlike the commonly used methionine‐ and choline‐deficient diet model 35, the mice on HFD gained weight, elevated circulating lipid levels, with the exception of triglycerides. Neither the weight gain nor elevated lipid levels were prevented by MD2 blockade, consistent with a finding on TLR4 knockout mice reported by Sutter *et al*. 10.

Several lines of evidence indicate that PPARγ has anti‐inflammatory properties 27, 28, 30, 36, 37 and moreover could limit the inflammatory injury in NALD‐NASH 25, 26. The anti‐inflammatory action of PPARγ has been linked to its ability to decrease expression of pro‐inflammatory molecules 29, 36. We observed that MD2 knockout or L6H21 significantly increased PPARγ expression in liver tissue and in HepG2 cells, suggesting that the protective effects of MD2 blockade could be attributed in part to the elevated PPARγ expression. The mechanism responsible for the increased PPARγ expression under MD2 blockade remains unclear but may be related to the TLR4‐regulated down‐regulation of PPARγ. Both signalling targets of activated TLR4, NF‐κB 30 and MAPK 38 can down‐regulate PPARγ, suggesting that under conditions of inhibited MD2‐TLR4 signalling, PPARγ down‐regulation was likewise inhibited.

In summary, the results indicated that MD2 inhibition with L6H21 was as effective as MD2 knockout in preventing the HFD‐induced hepatic lipid accumulation, pro‐fibrotic changes, expression of pro‐inflammatory molecules and MD2‐TLR4 complex formation in a mouse model of NASH. Moreover, these HFD‐associated inflammatory injuries were likely attributed at least, in part, to PA. Interestingly, MD2 knockout or L6H21 increased expression of PPARγ in liver tissue and the liver cell line, suggesting that PPARγ may contribute to the protective effects of MD2 blockade. Our results provide further evidence for the critical role of MD2 in the development of NASH. However, our study did not show the detailed molecular mechanism how MD2 blockage up‐regulated PPARγ. In addition, other molecule mechanisms may be also involved in MD2‐mediated liver lipid accumulation. We conclude that MD2 could be a potential therapeutic target for treatment of NAFLD/NASH, and the small molecule MD2 inhibitor, L6H21, was an effective and selective investigative agent for future mechanistic studies and pre‐clinical drug development.

## Conflicts of interest

No other potential conflicts of interest relevant to this article were reported.

## Supporting information


**Table S1**. The primer sequences of genes in real‐time qPCR assay.
