# Supplementary material for: Inhibition of MD2‐dependent inflammation attenuates the progression of non‐alcoholic fatty liver disease
Source: J Cell Mol Med. 2017 Oct 27;22(2):936–47. doi: 10.1111/jcmm.13395 (PMC5783870; doi:10.1111/jcmm.13395)
Supplement: Supplementary file 1 — Table S1. The primer sequences of genes in real‐time qPCR assay. [file JCMM-22-936-s001.docx]

***Supplementary information***

**Inhibition of MD2-dependent inflammation attenuates the progression of non-alcoholic fatty liver disease**

Yali Zhang^1,#^, Beibei Wu^2,#^, Xiangting Ge^2^, Shilong Ying^1^, Mengwei Hu^1^, Weixin Li^1^, Yi Huang^1^, Li Wang^1^, Xiaoou Shan^2,*^, Guang Liang^1,*^

**Table S1** The primer sequences of genes in real-time qPCR assay.

| Gene | Species | Forward Primer | Reversed Primer |
| --- | --- | --- | --- |
| TNF-α | Mouse | TGATCCGCGACGTGGAA | ACCGCCTGGAGTTCTGGAA |
| IL-1β | Mouse | ACTCCTTAGTCCTCGGCCA | CCATCAGAGGCAAGGAGGAA |
| ICAM-1 | Mouse | GCCTTGGTAGAGGTGACTGAG | GACCGGAGCTGAAAAGTTGTA |
| VCAM-1 | Mouse | TGCCGAGCTAAATTACACATTG | CCTTGTGGAGGGATGTACAGA |
| MCP-1 | Mouse | TCACCTGCTGCTACTCATTCACCA | TACAGCTTCTTTGGGACACCTGCT |
| COL-I | Mouse | TGGCCTTGGAGGAAACTTTG | CTTGGAAACCTTGTGGACCAG |
| COL-IV | Mouse | GCTCTGGCTGTGGAAAATGT | CTTGCATCCCGGGAAATC |
| α-SMA | Mouse | AAGAGCATCCGACACTGCTGAC | AGCACAGCCTGAATAGCCACATAC |
| TGF-β | Mouse | TGACGTCACTGGAGTTGTACGG | GGTTCATGTCATGGATGGTGC |
| MMP-9 | Mouse | TCTTCTGGCGTGTGAGTTTCC | CGGTTGAAGCAAAGAAGGAGC |
| PPAR-γ | Mouse | CCACTCGCATTCCTTTGACATC | TTGATCGCACTTTGGTATTCTTGG |
| β-actin | Mouse | CCGTGAAAAGATGACCCAGA | TACGACCAGAGGCATACAG |
| TNF-α | Human | CCCAGGGACCTCTCTCTAATC | ATGGGCTACAGGCTTGTCACT |
| IL-1β | Human | ACGCTCCGGGACTCACAGCA | TGAGGCCCAAGGCCACAGGT |
| ICAM-1 | Human | GAACCAGAGCCAGGAGACAC | TCCCTTTTTGGGCCTGTTGT |
| VCAM-1 | Human | GGCGCCTATACCATCCGAAA | TATGACCCCTTCATGTTGGC |
| MCP-1 | Human | AGAGACAGAAGAGCAACGGC | CGAGAACGAGCCTTGCAGTA |
| COL-I | Human | ATGTACCAGCCACTTCGTCC | TGGCTCCTGTAGATACGCCT |
| COL-IV | Human | CACAGCCAGACCATTCAG | AAGCGTTTGCGTAGTAATTG |
| α-SMA | Human | GACAATGGCTCTGGGCTCTGTAA | ATGCCATGTTCTATCGGGTACTTCA |
| MMP-9 | Human | ACGCACGACGTCTTCCAGTA | CCACCTGGTTCAACTCACTCC |
| PPAR-γ | Human | TCTCTCCGTAATGGAAGACC | GCATTATGAGACATCCCCAC |
| β-actin | Human | CCTGGCACCCAGCACAAT | GCCGATCCACACGGAGTACT |
